# Spectrum of Common and Rare Small Molecule Inborn Errors of Metabolism Diagnosed in a Tertiary Care Center of Maharashtra, India

**DOI:** 10.7759/cureus.27104

**Published:** 2022-07-21

**Authors:** Anish Tamrakar, Anjali Kale, Suvarna Magar, Ajay Kale, Vinod Ingale, Nilesh Shewale, Madhuri Engade, Madhavi Shelke

**Affiliations:** 1 Pediatrics, Mahatma Gandhi Missions (MGM) Medical College, MGM Institute of Health Sciences (IHS), Aurangabad, IND; 2 Pediatrics, Amrut Bal Rugnalay, Aurangabad, IND; 3 Pediatrics, Mahatma Gandhi Missions (MGM) Institute of Health Sciences (IHS), Aurangabad, IND; 4 Pediatrics, Pediatric Neurology, ICON Center (Integrated Center for Child Neurodevelopment), Aurangabad, IND

**Keywords:** small molecule metabolic diseases, inherited metabolic diseases, rare diseases, genetic diseases, inborn errors of metabolism

## Abstract

Introduction

Inborn errors of metabolism (IEM) form a large group of genetic diseases involving defects in genes coding for enzymes, receptors, and cofactors in the metabolic pathways of small and large molecules. The present study is the comprehensive data analysis of the tandem mass spectrometry (TMS) and urine metabolic pattern for the diagnosis of IEMs by gas chromatography and mass spectrometry (GC/MS) in samples received for high-risk IEM screening.

Methods

We conducted a retrospective analysis of children diagnosed with IEMs presenting at the genetic clinic of Mahatma Gandhi Missions (MGM) Medical College, Aurangabad. This article summarizes retrospective data of 40 pediatric cases over a three-year period, diagnosed with small molecule IEM based on the standard testing criteria.

Results

Out of 40, 17 patients (42.5%) were found to have organic acidemias, four (10%) had fatty acid oxidation defects, six (15%) had disorders of aminoacidopathies, seven (17.5%) had mitochondrial diseases, and three (7.5%) had urea cycle defects. One patient in each group (2.5% each) had carbohydrate metabolism defects, purine metabolic defects, and neurotransmitter metabolic defects.

Conclusions

This clinico-etiological profile study has thrown light on the clinical features and natural course of many common and rare IEMs, and it may provide clinicians with a deeper understanding of these conditions, allowing for improved early diagnosis and treatment of these diseases. Because of the high degree of consanguinity and marriages in the same community, common as well as many rare inherited metabolic diseases were diagnosed and novel genetic variants were identified.

## Introduction

Inborn errors of metabolism (IEM) form a large group of genetic diseases involving defects in genes coding for enzymes, receptors, and cofactors in the metabolic pathways of small and large molecules [1].

In most disorders, problems arise due to the accumulation of substances that are toxic or interfere with normal function, or symptoms arise due to the reduced ability to synthesize essential compounds. The majority of the IEMs are inherited in an autosomal recessive manner [1]. While individually rare, collectively, they are common, with an overall incidence of 1 in 2500 births [1]. More than 350 different IEMs have been described to date, and most of these are rare diseases/conditions [2]. It is well-documented that extended newborn screening with the use of tandem mass spectrometry (TMS) will prevent irreversible neurological damage and infant mortality [3]. Using relatively simple tests involving the detection of amino acids and acylcarnitines in dried blood spots on filter paper, TMS allows for the rapid screening and diagnosis of more than 40 metabolic disorders in amino acids, organic acids, and fatty acid oxidation, substantially improving the efficiency and accuracy of early diagnosis [4-5]. Further confirmation by urine gas chromatography and mass spectrometry (GCMS) helps in immediate therapeutic intervention and prevention of further morbidity [6]. Genetic testing has further helped to confirm the diagnosis by diagnosing cases even if TMS and GCMS results were negative or inconclusive but there was a strong suspicion of IEM. Prenatal testing in future pregnancies could only be offered based on a DNA-based diagnosis of an affected child.

The present study is a comprehensive data analysis of TMS and urine metabolic patterns for the diagnosis of IEM by GC/MS in samples received for high-risk IEM screening. Three patients were diagnosed based on only genetic testing and three were diagnosed based on typical MRI brain changes. We aimed to present an overview of various common and rare IEMs found in our region, and document the known and novel genetic variants in our population.

## Materials and methods

A total of 602 patients were screened over three years in the genetic clinic, out of which 112 patients were suspected of IEM. We have included data of 40 patients who were diagnosed as having small molecule IEM based on the TMS/GCMS test as the gold standard and genetic testing in a few of them.

This retrospective descriptive study was conducted at Mahatma Gandhi Mission Medical College in Aurangabad between October 2018 and September 2021. Children referred to the genetic clinic, MGM Hospital, Aurangabad, with features suggestive of IEM like unexplained neurological deterioration, persistent acidotic breathing, refractory convulsions, unexplained coma/focal deficits, unexplained hepatic presentations, recurrent vomiting with failure to thrive, and ketotic hypoglycemia were subjected to biochemical screening tests for IEMs like random blood sugar, serum electrolytes, serum lactate, arterial blood gases, serum ammonia, urine ketones, etc. If required, magnetic resonance imaging (MRI) of the brain was also done. If the result of the above screening test was suggestive of IEM, patients were further investigated with TMS and urine by GCMS. Blood and urine samples were collected on filter papers, air dried, and then sent to laboratories for analysis. Metabolites level more than the upper limit of the normal range on TMS and GCMS were taken as significant.

Clinical exome sequencing, a next-generation sequencing-based DNA test was advised to parents. A clinical exome study was done on 10 patients. Variants detected in the study were confirmed by the Sanger sequencing technique. Other tests that were utilized for diagnosis were plasma amino acids, urine or CSF amino acids, urine pterin assay, etc.

Inclusion criteria

Children aged between newborn to 18 years presenting at the genetic clinic of MGM Hospital and with a confirmed diagnosis of IEM were included in the study.

Exclusion criteria

Those with histories of meningitis, autoimmune diseases, and meningoencephalitis and proven other etiologies were excluded.

Data were collected by history taking, examination, and primary metabolic workup followed by biochemical and molecular testing. The age of presentation as a crisis, sex, consanguinity, clinical signs, and symptoms during the presentation, biochemical, molecular, MRI brain findings, diagnosis, and course in the ward in terms of death or discharge were noted.

We also performed a carrier status of parents when a proband sample was not available for molecular diagnosis. IEMs were categorized as amino acid disorders; fatty acids disorders; organic acid disorders; mitochondrial disorders; carbohydrate metabolic disorders; neurotransmitter disorders; and purine and pyrimidine disorders.

The Institutional Ethics Committee approval number was ECRHS/2019/58. Categorical data were expressed as numbers and percentages.

## Results

Out of 602 patients referred to the genetic OPD, 40 patients were diagnosed with small molecule IEMs (6.6%). TMS and GCMS were performed in 112 patients, out of which 34 patients were diagnosed with some IEM. Among 40 patients, six patients were diagnosed based on MRI brain and/or genetic testing.

Thirty-five patients (87.5%) were below two years of age, and five patients (12.5 %) were more than two years of age. Twenty-eight patients (70%) were males, and 12 patients (30%) were females. Parental consanguinity was seen in 29 patients (72.5%). Positive family history or a previous death of a sibling was seen in nine patients (22.5%) (Table 1).

**Table 1:** Demographic characteristics

Demographic characteristics	No. of Patients (n)	Percentage (%)
Age		
< 2 year	35	87.5
> 2 years	5	12.5
Gender		
Male	28	70
Female	12	30
Consanguinity		
Yes	29	72.5
No	11	27.5
Siblings affected		
Yes	9	22.5
No	31	77.5

Acute encephalopathy was a presenting clinical feature in 21 (52.5%) patients, which was followed by seizures as the presenting complaint in 19 (47.5 %) patients. Global developmental delay (GDD) and recurrent vomiting were seen in nine patients each (22.5%), hypoglycemia in five patients (12.5%), and hepatic failure in three patients (7.5%) (Table 2).

**Table 2:** Common clinical presentations of IEMs IEM: inborn errors of metabolism

Symptoms/signs	No. of Patients (n)	Percentage (%)
Acute encephalopathy	21	52.5
Global developmental delay	9	22.5
Seizure disorder	19	47.5
Hepatic failure	03	7.5
Hypoglycemia	05	12.5
Recurrent vomiting	09	22.5

Organic aciduria was diagnosed in 17 patients (42.5%), four patients (10%) had fatty acid oxidation defects, six (15%) had disorders of aminoacidopathies, seven (17.5 %) had mitochondrial diseases, and three (7.5 %) had urea cycle defects. Metabolic defects of carbohydrates, purine, and neurotransmitters were diagnosed in one patient each, respectively (2.5% each) (Table 3). 

**Table 3:** Disease spectrum of different types of inborn errors of metabolism Phenylketonuria: PKU; lysin uric protein intolerance: LPI; propionic aciduria: PA; glutaric aciduria II: GA2; methylmalonic aciduria: MMA; glutaric aciduria type I: GA1; maple syrup urine disease: MSUD; medium chain acyl CoA dehydrogenase deficiency: MCAD deficiency; long chain 3 hydroxy acyl CoA dehydrogenase deficiency: LCAD deficiency; MRS: magnetic resonance spectroscopy

Case. No.	Diagnosis	Age/Sex	Presentation	Baseline investigation	Key Biochemical findings in TMS	Urine GCMS
1, 2	Hyperphenylalanemia/Phenylketonuria(PKU)	9 months/female, 6 months/male	GDD (global developmental delay)	-	↑ Phenylalanine	↑ 2 hydroxy phenyl lactic acid, ↑ 3 phenyl lactic acid
3	Lysin uric protein intolerance (LPI)	3 years 3 months/male	recurrent infections, hepatosplenomegaly, bicytopenia	-	Negative	↑ lysine 3, ↑ Lysine 4
4, 5	Tyrosinemia type I	1 year 3 months/male, 15 days/male	hepatosplenomegaly, liver failure	Hyperammonemia	↑ tyrosine,	↑ succinyl acetone, ↑ phenyllactic-2, ↑4-OH phenylacetic-2
6	Hyperhomocysteinemia	3 months/female	GDD	Metabolic acidosis, lactic acidosis, hyperammonemia	↑ C3	↑ methylmalonic acid, ↑ homocysteine
7	2 methyl 3 hydroxy butyric aciduria	7 months/female	encephalopathy, ketonuria	Metabolic acidosis, lactic acidosis, hyperammonemia	↑ tiglylcarnitine (C5:1), ↑ Malonyl carnitine/Hydroxy butyryl carnitine-C3DC/C4OH	↑ 2 methyl 2 hydroxybutyric acid, 2 ↑ tiglyglycine
8	2- hydroxy glutaric aciduria	1 year 5 months/female	Encephalopathy	Metabolic acidosis, lactic acidosis, hyperammonemia	Negative	↑ 2- hydroxy glutaric acid, ↑ glutaric acid
9, 10, 11	Propionic aciduria (PA)	1 year 4 months/male, 15 days/male, 10 days/male	Encephalopathy	Metabolic acidosis, lactic acidosis, hyperammonemia	↑ leucine/isoleucine/hydroxyproline, ↑ valine, ↓ free carnitine, ↑ C3-12.	↑ 3 hydroxy propionic acid-2, ↑glutaric acid 2, ↑ methyl citric acid-4
12	Oxoprolinuria	2 months/male	encephalopathy	Metabolic acidosis, lactic acidosis, hyperammonemia	Negative	↑ 5- oxoproline-2
13, 14, 15	Glutaric aciduria II (GA2)	4 months/male, 10 years/female, 13 months/male	encephalopathy	Metabolic acidosis, lactic acidosis, hyperammonemia	↑ C8 and alanine, ↓ free carnitine	
16	Methylmalonic aciduria (MMA)	10 months/female	encephalopathy	Metabolic acidosis, lactic acidosis, hyperammonemia	↑ C3	↑ methylmalonic acid-2
17	Glutaric aciduria type I (GA1)	5 months/male	Convulsion, encephalopathy	Metabolic acidosis, lactic acidosis, hyperammonemia	↑ glutaryl carnitine	
18	3 methyl glutaconic aciduria type 5	2 years 3 months/ male	GDD	Metabolic acidosis, lactic acidosis, hyperammonemia	Negative	↑ lactate, ↑ 2 OH butyric acid, ↑ 3 OH butyric acid 2, ↑ adipic 2(C6), C8
19	Biotin-responsive basal ganglia disease	2 years/male	Convulsions, altered sensorium, encephalopathy	Lactic acidosis	Negative	Negative
20	HMG CoA lyase deficiency	21 days/male	convulsions, encephalopathy	Metabolic acidosis, lactic acidosis, hyperammonemia	↑ methyl malonyl carnitine, ↑ propionyl carnitine	↑3 OH methylglutaconic acid, ↑ 3 OH isovaleric acid
21, 22	Maple syrup urine disease (MSUD)	9 days/female, 16 months/male	Encephalopathy	Metabolic acidosis, lactic acidosis, hyperammonemia	↑ leucine, ↑ valine	
23	Methylmalonic aciduria (MMA)	3 days/male	Encephalopathy	Metabolic acidosis, lactic acidosis, hyperammonemia	↑propionyl carnitine, ↑C3/C2 ratio, ↑ C3/C6 ratio	
24, 25, 26	Citrullinemia type I	8 days/male, 3 years 10 months/male, 8 days /female	Convulsions, metabolic encephalopathy	Hyperammonemia	↑ citrulline, ↑ citrulline/arginine ratio	
27	3 OH acyl CoA dehydrogenase deficiency	3 months/male	Distension of the abdomen, vomiting, difficulty in breathing	Hypoglycemia, metabolic acidosis	↓ alanine, ↓methionine, ↓ ornithine, ↑ argino succinic acid, ↓ glutamic acid, ↑ malonylcarnitine	↑ hydroxyadipic acid-3, ↑ glycerol 3 phosphate-4
28	Medium chain acyl CoA dehydrogenase deficiency (MCAD deficiency)	6 months/female	Motor developmental delay	Hypoglycemia, metabolic acidosis	↑ C6 and C8	↑ C6, ↑ C8, ↑ C10, ↑ C12
29, 30	Long chain 3 hydroxy acyl CoA dehydrogenase deficiency (LCAD deficiency)	5 months/male, 2 years/male	Global developmental delay, seizures, encephalopathy	Hypoglycemia, metabolic acidosis	↑ C16 OH, ↑ C18OH, ↑ Ornithine, ↑ Alanine	↑ C6, ↑ C8, ↑ C10, ↑C12, and their corresponding 3 hydroxy dicarboxylic acids
31, 32, 33	Carnitine uptake defect	2 months/male, 9 months/male, 7 years/female	Yellowish discoloration of the body, decreased activity	Recurrent hypoglycemia, metabolic acidosis	↓ free, ↓ acetylcarnitine, ↓ butyryl carnitine	
34, 35, 36	Mitochondria disease, Leigh disease based on MRI brain and MRS	2 years/male, 18 months/male, 3 years/male	GDD, convulsions, encephalopathy	Hyperlactatemia	negative	
37	Mitochondria DNA depletion syndrome – 7	2 years/female	Motor developmental delay, vision loss, fundus shows optic atrophy	Hyperlactatemia	negative	
38	Succinic semialdehyde dehydrogenase deficiency	5 months/female	GDD with hypotonia	-		↑ 2 deoxytetronic acid, ↑ 4 hydroxybutyric acid
39	Inosine triphosphate phosphohydrolase deficiency	17 months/male	GDD, convulsions, encephalopathy, microcephaly, hypotonia	-	negative	negative
40	Classical galactosemia	4 months/ Female	Yellowish discoloration of body, distension of abdomen, clay color stools	Hypoglycemia, increased liver enzymes, and prothrombin time	↑ arginine, time-resolved fluoro immunoassay –, ↑ T GAL	↑ galactitol-, ↑ glycerol 3 phosphate

Results of the molecular analysis are shown in Table 4.

**Table 4:** Molecular analysis of study participants

Case no.	IEM presentation	Gene	Nucleotide Variant	Amino Acid Variant	Type of Mutation	Type of Variant	Pathogenicity	Minor Allele Frequency in gnomAD
2	Lysinuric protein intolerance	SLC7A7	c.110 dupT	p.Ser38LeufsTer4	Homozygous	Frameshift	LP	Not detected, novel
3	Tyrosinemia type 1	FAH	c.192 G >T	p.Gln64His	Homozygous	Missense	P	0.000036
4	Hyperphenylalaninemia BH4-deficient, A	PTS	c.200C>T	p.Thr67Met	Homozygous	Missense	P	0.0000131
17	3 methylglutaconic aciduria type 5	DNAJC19	c.250C>T	p.Arg84Ter	Homozygous	Nonsense	LP	0.0000199
18	Biotin-responsive basal ganglia disease	SLC19A3	c.595 T>A	p.Phe199Ile	Homozygous	Missense	LP	Not detected/Novel
20	Maple syrup urine disease	BCKDHA	c.1251delC	p.Gln418SerfsTer66	Homozygous	Frameshift	LP	Not detected/ Novel
22	Methylmalonic aciduria	MMUT	c.643G>T c.692dup	p.Gly215Cys p.Tyr231Ter	Compound heterozygous	Missense Nonsense	P P	Novel 0.0000131
23	Maple syrup urine disease	BCKDHA	c.868 G>A	p.Gly290Arg	Homozygous	Missense	P	0.0000397
26	Citrullinemia type I	ASS1	c.1168G>A	p.Gly390Arg	Homozygous	Missense	P	0.0000263
35	Mitochondrial DNA depletion syndrome 7	TWNK	c.1003C>A c.2050A>C	p.Pro335Thr	Compound heterozygous	Missense Missense	LP VUS	Not detected/Novel 0.000092
38	Succinic semialdehyde dehydrogenase deficiency	ALDH 5A1	c.701 C>T	p.Pro234Leu	Homozygous	Missense	LP	Not detected/Novel
39	Inosine triphosphate phosphohydrolase deficiency	ITPA	c.137delA	p.Gln46ArgfsTer43	Homozygous	Frameshift	LP	Not detected/Novel
40	Classical galactosemia	GALT	c.142 C>T c.610 C>T	p.Arg48Cys p.Arg204Ter	Compound heterozygous	Missense Missense	VUS P	0.000000657 0.00000065

Next-generation-based clinical exome sequencing technology was used for molecular diagnosis in 13 patients. Genomic DNA from the peripheral blood sample or dry blood spot was enriched for the complete coding regions and splice site junctions of genes included in the clinical exome panel. Paired-end sequencing was performed on an Illumina platform (Illumina, San Diego), California. Data were filtered and analyzed to identify variants of interest and interpreted in the context of a single most damaging, clinically relevant transcript to report. The latest population frequencies or minor allele frequencies (MAF) of variants were checked in the gnomAD exome database [7]. Minor allele frequency of each variant was suggestive that the variants were extremely rare and Case Nos. 2, 18, 20, 35, 38, and 39 had novel variants not detected previously as per the gnomAD database. All the variants in our study are pathogenic or likely pathogenic except for one variant each in Case Nos. 35 and 40, which was a variant of uncertain significance. The latest information of all the variants was also rechecked on the Varsome database accessed on 26/06/2022 and Table 4 includes the latest information about all the variants [8].

## Discussion

We present the cases of IEM referred to the genetic clinic from the pediatric and neonatal intensive care, wards, and OPD. The most common reason for referral was metabolic encephalopathy, followed by global developmental delay and seizure disorder, with the less common being hypoglycemia, hepatic failure, etc. An IEM could potentially be underdiagnosed and a high index of suspicion and team effort is essential to diagnose IEM. The availability of advanced biochemical testing helped in the definitive diagnosis. An article on the testing modalities of IEMs has helped us reach the diagnoses [9]. Metabolite pattern recognition in all the tests helps arrive at a specific diagnosis. TMS, GCMS, and HPLC of amino acids in blood and urine are the most common diagnostic modalities to aid in the definitive diagnosis of small molecule IEM [10].

The most common IEM group in our study was organic acidemias, accounting for 42% of the total IEM. Aminoacidopathies, organic aciduria, and urea cycle disorders (UCD) are known to present as metabolic encephalopathy and hence metabolic encephalopathy was the most common symptom in our study. Among organic acidurias, glutaric aciduria II (GAII), propionic aciduria (PA), methylmalonic aciduria (MMA), glutaric aciduria I (GA-I), MSUD, biotin-responsive basal ganglia disease were the common organic acidurias that can be picked up on extended newborn screening. A pilot study in India also identified these as common organic acidurias in newborn screening [11].

The rare organic acidurias diagnosed were 2-methyl-3-hydroxybutyric aciduria, 2-hydroxy glutaric aciduria, oxoprolinuria, 3-methylglutaconic aciduria type 5, HMG CoA lyase deficiency, riboflavin deficiency, etc. A seven-month-old girl child with metabolic encephalopathy was diagnosed with 2-methyl-3-hydroxybutyric aciduria, which is an X-linked dominant rare IEM and is not reported in India. A two-year-old male child with global developmental delay was diagnosed with 3 methylglutaconic aciduria type 5 also called dilated cardiomyopathy and ataxia (DCMA) based on homozygous likely pathogenic variants in exon 5 of the DNAJC19 gene c.250C>T (pArg84Ter). It was a frameshift variant, and this disease is not reported in India. To date, the maximum cases of DCMA reported involve individuals from the Dariusleut Hutterite population, an endogamous population of the Great Plains region of Canada and the northern United States. Update on cases, natural history, and phenotypes in this disease have been described by Machiraju et al. and is matching with the case [12]. This patient had a global developmental delay with ataxia and micropenis requiring testosterone injections in infancy. Two-dimensional echo was normal. Another two-year-old child with global developmental delay, movement disorder, and seizure disorder was TMS and GCMS negative but exome sequencing revealed homozygous likely pathogenic variants in the SLC19A3 gene in exon 3, c.595 T>A causing biotin responsive basal ganglia disease. This variant is not detected in the ClinVar and GnomAD databases and is a novel variant. Our patient had a similar phenotype as that of the cases in the study by Alfadhel et al. [13]. A nine-day-old female succumbed to metabolic encephalopathy and TMS was screened positive for MSUD. Carrier screening of parents by next-generation sequencing revealed that both parents were carriers of a heterozygous likely pathogenic variant in the BCKDHA gene in exon 9 c.1251delC. This was a novel variant not reported in the literature previously. There is a case series by Narayan et al. from India, which identified severe MSUD due to mutations in the BCKDHA gene than the BCKDHB gene [14]. Both our patients also have severe presentation and variants in the BCKDHA gene [11]. BCKDHA gene mutations in the study by Bashyam et al. [15] showed mutations in exons 8 and 9, and our patients had mutations in exons 7 and 9. A three-day-old male child succumbed to encephalopathy that also had similar sibling death on Day 3 of life, and was screened positive for methylmalonic aciduria (MMA). His parents were having compound heterozygous likely pathogenic variants in the MMUT gene c.643G>T (pGly215Cys) and a second variant c.692dup, pTyr231Ter. Observed variants are already reported in the literature and are severe mutations or mut0 phenotypes with no enzyme activity [16-17].

Among the common aminoacidopathies, two patients were diagnosed with tyrosinemia types 1 and 2 with hyperphenylalaninemia, one with hyper-homocysteinemia, and one with a rare amino acid disorder of lysinuric protein intolerance (LPI). We have already reported the case of LPI, which usually presents as a hyperammonemia encephalopathy crisis, but our patient presented with hepatosplenomegaly and bicytopenia. The patient is on lysine, citrulline, and arginine supplements with adequate growth and reduced liver and spleen size [18]. The variant c.110 dupT in SLC7A7 is a novel variant causing LPI. One patient with hyperphenylalaninemia underwent a urinary pterin assay and was suspected to be suffering from a biopterin pathway defect. His clinical exome analysis revealed a homozygous likely pathogenic variant in exon 4, c.200C>T (pThr67Met) in the PTS gene, causing hyperphenylalaninemia, BH4-deficient, A. There is a case series from India on BH4-deficient hyperphenylalaninemia, and it reported one patient with a PTS gene mutation and a similar phenotype as seen in our patient [19].

Among urea cycle defects, all three patients were diagnosed with citrullinemia, two patients were diagnosed at a neonatal age with encephalopathy, and the third child was diagnosed late with behavioral changes. Citrullinemia was a common urea cycle defect in the latest study from India. Molecular analysis in one patient revealed a common mutation of c.1168G > A (pGly390Arg) as described in the study by Bijarniya et al. [20].

Among the fatty acid oxidation defects, the acylcarnitine profile in TMS was suggestive of carnitine uptake defect in three patients, two patients with long chain acyl CoA dehydrogenase deficiency (LCAD), and one each with a short chain and medium chain acyl CoA dehydrogenase deficiency (SCAD and MCAD). Patients with carnitine uptake defects, and MCAD deficiency presented with symptoms of hypoglycemia, hepatomegaly, raised liver enzymes, and deranged PT international normalized ratio (INR). All patients are under treatment with a reduced episode of hypoglycemia following precautionary advice. The study by Singh et al. and Mahale et al. have demonstrated the diagnosis of FAODs by TMS [21-22]. Patients with SCAD and LCAD deficiency presented with metabolic encephalopathy with a seizure disorder and succumbed to acute encephalopathy. A study by Vengalil et al. presented 19 patients with dropped head syndrome and myopathy [23].

One patient was diagnosed with a neurotransmitter metabolic defect of gamma amino butyric acid (GABA), a girl child with global developmental delay and autistic features. She was diagnosed with succinic semialdehyde dehydrogenase deficiency with a c.701 C>T homozygous likely pathogenic variant in the ALDH5A1 gene. Approximately 450 cases are diagnosed with SSADH deficiency worldwide [24].

One child was diagnosed with a purine metabolism defect. A 17-month-old child with a global developmental delay was diagnosed with an inosine triphosphate phosphohydrolase deficiency based on homozygous pathogenic variants in the ITPA gene, c.137delA (pGln46ArgfsTer43). Homozygous or compound heterozygous mutations in the ITPA gene are known to cause neurological presentations. One case reported by Karthik et al. has similar features of encephalopathy, global developmental delay, and MRI brain abnormalities [25]. It is a novel variant not detected in the gnomAD or ClinVar database.

TMS and GCMS were screened positive for galactosemia in the case of a two-month-old girl child who presented with hepatic failure, hypoglycemia, and bilateral cataract. Her DNA study revealed compound heterozygous variants. Pathogenic variant c.610 C>T and a variant of uncertain significance c.142 C>T were detected in the GALT gene. Variant c.610 C>T is already reported in the literature and variant c.142 C>T is predicted damaging by more than three pathogenicity prediction tools [8]. A study by Singh et al. has described the predominant Duarte 2 variant indicating milder galactosemia in India though our patient had severe galactosemia [26].

Among the four patients with mitochondrial disease, all four had typical MRI brain findings of symmetrical T2-weighted hyperintensities in the bilateral, basal ganglia, midbrain, pons, and cerebellum, and a lactate peak on magnetic resonance spectroscopy (MRS). A two-year-old girl with motor delay and optic atrophy, with TMS negative, underwent molecular testing and was found to have compound heterozygous variants in the TWNK gene. One heterozygous likely pathogenic variant c.1003 C>A and another variant of unknown significance c.2050 A>C in the TWNK gene causing mitochondrial DNA depletion syndrome 7 or infantile-onset spinocerebellar ataxia (IOSCA) were detected. The founder variant in the TWNK gene has been identified in the genetically isolated population of Finland only, where IOSCA is the second-most common inherited ataxia [27]. Other TWNK variants and their different phenotypic expressions have been described in affected individuals in a study by Faruq et al. [28].

In developed countries, newborn screening is being done widely for varying metabolic disorders. The conditions screened are six, 29, and 23 conditions in the UK, USA, and Australia, respectively [29]. Among the patients who presented for the first time, more than 50% of patients could have been potentially picked up by NBS.

Molecular testing was done in 13 cases (32.5%) of the study cohort. We could not confirm using molecular analysis in a higher proportion and had to do parental carrier screening by clinical exome studies in a few. As all the conditions in the present study are inherited in an autosomal recessive manner, we got 72% (8/11) of a pathogenic variant in homozygous status and only 23% (3/13) as compound heterozygous. In the present genomic era, next-generation sequencing is the most common test utilized to detect single gene disorders. Exome sequencing is an effective technology for diagnosing metabolic disorders [30]. Molecular analysis has not only helped the index child but also helped in the prenatal diagnosis of three families and fetuses that were carriers of the disease.

The strengths of our study are a detailed description of biochemical testing aiding in the definitive diagnosis and diagnosing of rare IEMs and unique and novel variants of rare IEMs. TMS urine GCMS is a cost-effective strategy for diagnosing IEMs, which is well-utilized in our study.

The weakness of our study is that we could not confirm genetic variants in many patients. We could have missed actual IEM cases that were initially screened negative because of late sampling done at the time of recovery when metabolites disappear to be detectable by TMS and GCMS.

## Conclusions

Because of the high degree of consanguinity and marriages in the same community, common, as well as many rare, inherited metabolic diseases were diagnosed. The clinico-etiological profile study has thrown light on clinical features and the natural course of many common and rare IEMs, and it may provide clinicians with a deeper understanding of these conditions, allowing for improved early diagnosis and treatment of these diseases. The rare IEMs and novel variants in this study have added valuable information to the genetic data pool.
